# Mechanisms of LTR-Retroelement Transposition: Lessons from *Drosophila melanogaster*

**DOI:** 10.3390/v9040081

**Published:** 2017-04-16

**Authors:** Lidia N. Nefedova, Alexander I. Kim

**Affiliations:** Department of Biology, Moscow State University, Moscow 119992, Russia; lidia_nefedova@mail.ru

**Keywords:** *Drosophila*, LTR-retrotransposon, errantivirus, retrovirus, transposition

## Abstract

Long terminal repeat (LTR) retrotransposons occupy a special place among all mobile genetic element families. The structure of LTR retrotransposons that have three open reading frames is identical to DNA forms of retroviruses that are integrated into the host genome. Several lines of evidence suggest that LTR retrotransposons share a common ancestry with retroviruses and thus are highly relevant to understanding mechanisms of transposition. *Drosophila melanogaster* is an exceptionally convenient model for studying the mechanisms of retrotransposon movement because many such elements in its genome are transpositionally active. Moreover, two LTR-retrotransposons of *D. melanogaster*, *gypsy* and *ZAM*, have been found to have infectious properties and have been classified as errantiviruses. Despite numerous studies focusing on retroviral integration process, there is still no clear understanding of integration specificity in a target site. Most LTR retrotransposons non-specifically integrate into a target site. Site-specificity of integration at vertebrate retroviruses is rather relative. At the same time, sequence-specific integration is the exclusive property of errantiviruses and their derivatives with two open reading frames. The possible basis for the errantivirus integration specificity is discussed in the present review.

## 1. Introduction

Unlike the human genome in which retrotransposons occupy more than 65% of the genome [1], in *Drosophila melanogaster*, retrotransposons accounts for only approximately 5% of the genome [2]. Nevertheless, *D. melanogaster* is an exceptionally convenient model for studying the mechanisms of retrotransposon movement because many of the retrotransposons in its genome are transpositionally active (in contrast to human retrotransposons) [3].

Retrotransposons that have long terminal repeat (LTR retrotransposons) occupy a special place among *D. melanogaster* retrotransposons. LTR retrotransposons have a varying structure and can contain different open reading frames (ORFs): from one to three. Most complex forms are retrotransposons that contain three ORFs: ORF1 (corresponding to the *gag* gene of retroviruses) encodes capsid proteins, ORF2 (*pol*) encodes protease, reverse transcriptase, RNase H, and integrase, and ORF3 (*env*) encodes a product that is responsible for cell receptor recognition and virus penetration into the cell. Thus, the structure of LTR retrotransposons that have three ORFs is identical to DNA forms of vertebrate retroviruses that are integrated into the host genome. Not by chance, two LTR retrotransposons of *D. melanogaster*, *gypsy* and *ZAM*, have been found to have infectious properties and have been classified as endogenous retroviruses [4,5].

**Figure 1:**
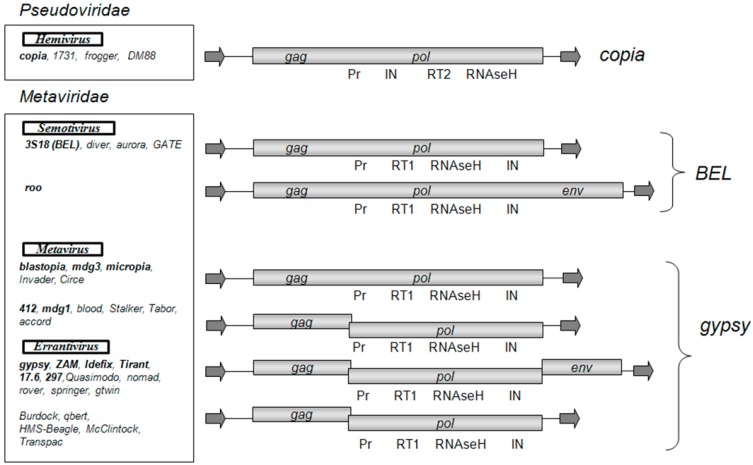
Structural organization and classification of *Drosophila melanogaster* long terminal repeat (LTR) retrotransposons. As shown: open reading frames (*gag*, *pol*, *env*) and the Pol domains (Pr, protease; RT1 and RT2, reverse transcriptase types 1 and 2; IN, integrase). The arrows indicate LTRs. LTR retrotransposons are distributed in groups and genera according to the phylogenetic analysis conducted in [9]. The LTR retrotransposon families introduced by the International Committee on Taxonomy of Viruses (ICTV) in *Metaviridae* and *Pseudoviridae* are highlighted in bold.

According to the classification of LTR retrotransposons, which is based on a comparative analysis of the conserved domain of reverse transcriptases, there are three groups of LTR retrotransposons that correspond to the individual phylogenetic clades: Gypsy, Copia, and BEL [6]. The *D. melanogaster* genome contains 36 families of LTR retrotransposons [7]. The BEL and Copia groups are represented by five and four families, respectively, and the Gypsy group is represented by 27 families of retrotransposons. While the LTR retrotransposons of the Copia and BEL groups have one ORF, the Gypsy group is heterogeneous in composition and is represented by LTR retrotransposons with one, two, or three ORFs (Figure 1). The observed diversity of the Gypsy group of LTR retrotransposons obviously shows the recent origin of the currently existing families and transposition activity of the retrotransposons in this group. A high level of polymorphism is observed not only between families of the group but also within some families of the group, e.g., within the family of *gypsy* [7]. There are 12 subfamilies in *gypsy* (*gypsy1*–*gypsy12*), which are polymorphic in sequence and length (the difference can be up to 1000 base pair; bp). The presence of such a large number of subfamilies indicates that *gypsy* has the highest rate of diversification in the *D. melanogaster* genome. 

Some *D. melanogaster* LTR retrotransposons are included in the international classification of viruses (ICTV) [8], in the *Metaviridae* family, which includes 3 genera: *Errantivirus* (includes six families of the Gypsy group of LTR retrotransposons with three ORFs: *gypsy*, *ZAM*, *Idefix*, *Tirant*, 297, and *17.6*), *Metavirus* (includes five families of the Gypsy group of LTR retrotransposons with one and two ORFs: *412*, *mdg1*, *mdg3*, *blastopia*, and *micropia*), and *Semotivirus* (includes two families of the BEL group of LTR retrotransposons: *roo* and *3S18*). The mobile element *copia* was assigned by the ICTV to the *Hemivirus* genus of the *Pseudoviridae* family.

According to the phylogenetic analysis, which is based on a comparative analysis of *gag* and *pol* ORFs, all Gypsy group LTR retrotransposons can be classified either as genus *Metavirus* or *Errantivirus*. *Metavirus* contains LTR retrotransposons that are divided into two separate subgroups: blastopia (with one ORF) and 412 (with two ORFs) (Figure 1). The LTR retrotransposons with two ORFs, *McClintock*, *qbert*, *accord*, *Burdock*, *HMS-Beagle*, and *Transpac*, are derived from errantiviruses and have lost their infectious properties [9]. Therefore, these LTR retrotransposons that have two ORFs should be classified to the *Errantivirus* genus.

Retroviruses of vertebrates belong to the *Retroviridae* family, which is divided into two subfamilies (*Orthoretrovirinae* and *Spumaretrovirinae*) that include six genera (*Alpha-*, *Beta-*, *Delta-*, *Gamma-*, *Epsilonretrovirus* and *Lentivirus*), and one genus (*Spumavirus*), respectively [8]. According to the phylogenetic analysis of Gag and Pol sequences, the *Retroviridae* family can be divided into three classes [10]. Class 1 includes *Gamma*- and *Epsilonretrovirus*, class 2 includes *Lentivirus*, *Alpha*-, *Beta*-, and *Deltaretrovirus*, and class 3 includes *Spumaretrovirus* and endogenous retroviral (ERV) elements. For a number of structural features, the same phylogenetic analysis shows that *D.melanogaster* retrotransposons of the 412 subgroup of the *Metavirus* genus are similar to class 1 retroviruses. This also shows that retrotransposons in the genus of the blastopia subgroup of *Metavirus* are similar to class 2 retroviruses, and that errantiviruses are similar to class 3 retroviruses [11]. Thus, vertebrate retroviruses and LTR retrotransposons/retroviruses of *Drosophila* have a common evolutionary history and should be considered in parallel. Because the *D. melanogaster* genome has a large variety of LTR retrotransposons and retroviruses, we can use this organism as a model to generally analyze the evolutionary mechanisms of retroelement transposition in eukaryotes.

## 2. Errantiviruses Specifically Integrate into the Target DNA

**Figure 2:**
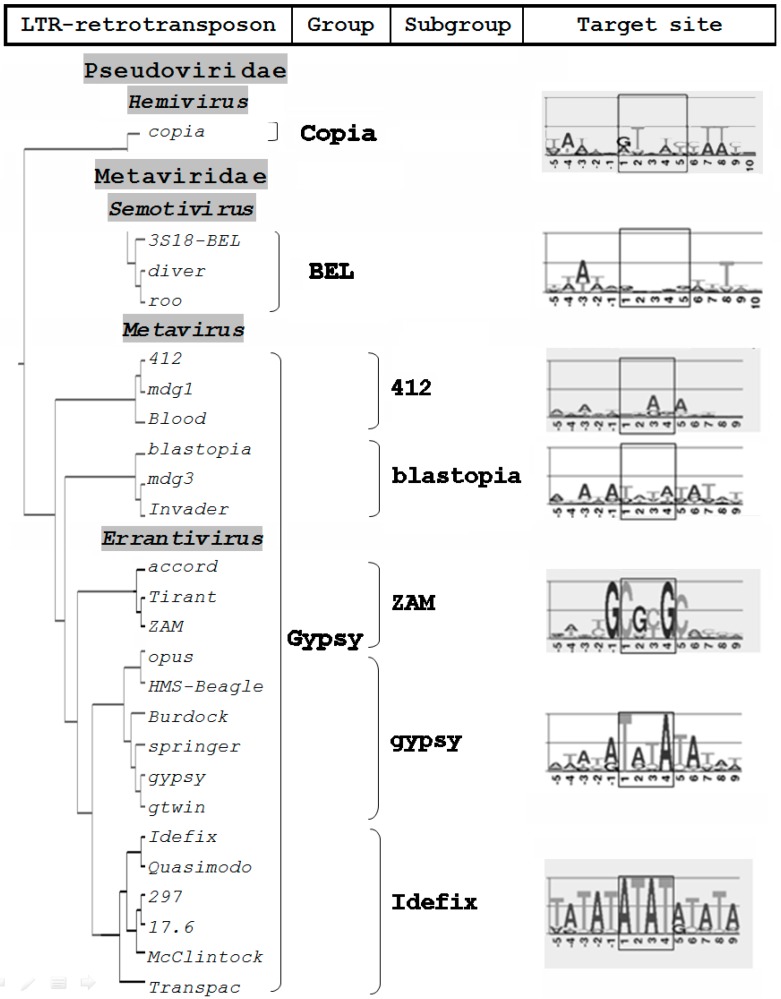
Integration sites of *D. melanogaster* LTR retrotransposons. A phylogenetic tree construction is based on a comparison of the amino acid sequences of the integrases of *D. melanogaster* LTR retrotransposons [25]. Visualization of the target site duplication was made using WEBLOGO (version 3) [29].

There is still no clear understanding of the specificity of retroviral integration within the target site. The efficiency of integration primarily depends on the efficiency of the integrase enzyme. The interaction of integrase with tethering factors is the basis of integration targeting, at least for murine leukemia virus (MLV) and human immunodeficiency virus (HIV) retroviruses and yeast retroelements Ty1, Ty3 and Ty5 [12,13,14,15,16]. On the other hand, the target structure can contribute to the targeted integration. It is believed that the choice of the target DNA can affect a variety of factors, including the transcriptional status of DNA, methylation, association of DNA with histones and other DNA-binding proteins, DNA bending, etc. [17,18,19,20]. Furthermore, the strict specificity of integration is not characteristic of vertebrate retroviruses [21,22,23,24]. During the analysis of the retroviral integration sites of HIV-1, a “weak” target site consensus, GT(A/T)AC, was found [23]; it is similar to the target of the *D. melanogaster* Copia group LTR retrotransposons (Figure 2).

It was found that errantiviruses and their derivatives that have two ORFs exhibit a specificity of choice for the target DNA. These LTR retrotransposons can be divided into three subgroups, *gypsy*, *ZAM*, and *Idefix*, the representatives of which have a different specificity for the target [25]. In all three cases, the more frequent target is a palindromic (or imperfect palindromic) sequence: TATA, CGCG, or ATAT (Figure 2). More recent studies, using population genomic resequencing data from hundreds of strains of *D. melanogaster* as well as computational analyses, reveal the same specific target site preferences of *D.melanogaster* retroelements [26,27,28].

The specificity of integration is an exclusive property of errantiviruses and derived LTR retrotransposons that have two ORFs. The fact that the sequence motifs at errantivirus target sites are always palindromes is quite remarkable. Recent results indicate that vertebrate retrovirus integration sites contain a shared non-palindromic motif [30]. The shared motif is 5′-T(N1/2) [C(N0/1)T|(W1/2)C]CW-3′, where the square brackets represent the duplicated region, W denotes A or T, and | represents the axis of symmetry.

## 3. Repeats in the 5′-UTR Can Direct Heterochromatic Localization of Errantiviruses

It is noteworthy that ZAM subgroup errantiviruses integrate preferentially into GC-rich repeats. According to data in FlyBase [31], *ZAM* errantivirus insertions were found only in the constitutive heterochromatin, and *Tirant* insertions were found only in the euchromatin and facultative heterochromatin in the reference *Drosophila* genome. The other LTR retrotransposons were found both in euchromatin and heterochromatin. Of note, both *ZAM* and *Tirant* have tandem repeats in the 5′-untranslated region (5′-UTR): the number of repeats in *ZAM* 5′-UTR is 2.3 repeats (each one is 307 bp in length), and the number of repeats in *Tirant* 5′-UTR varies from two to six (each one is 102 bp). The role of the repeats in the *Tirant* 5′-UTR is still unclear. Previously, it was shown that repetitive sequences in the 5′-UTR *ZAM* errantivirus that are phylogenetically similar to *Tirant* interact with the heterochromatin protein, HP1a, which probably directs its heterochromatic localization [32].

**Figure 3:**
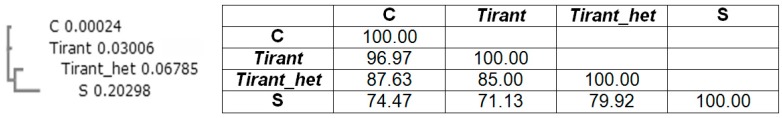
Phylogenetic tree of the 5’-untranlsated region (5′-UTR) repeat module (102 base pair; bp) in *Tirant* of *D. simulans* (subfamilies S and C) and *D. melanogaster* (subfamilies *Tirant* and *Tirant_het*) and sequence identity matrix (%).

Earlier in the *Drosophila simulans* genome, two subfamilies of *Tirant* were found: C-euchromatic (found both in *D. simulans* and *D. melanogaster*) and S-heterochromatic (found only in *D. simulans*) [33,34]. Localization of each subfamily in a certain type of chromatin was determined via association with modified histones, H3K9me2, H3K4me2, and H3K27me3, which are epigenetic markers of constitutive heterochromatin, facultative heterochromatin, and euchromatin, respectively. *Tirant* primarily associates with facultative heterochromatin [34]. By analyzing the heterochromatin component of the sequenced genome, we discovered a new heterochromatin subfamily of *Tirant* that consists of four copies and named this subfamily *Tirant_het*. The *Tirant_het* subfamily is not the same as the S-subfamily and represents an older, now non-functional, individually evolving heterochromatic branch of the ZAM subgroup of the Gypsy group. It contains two repeat modules in the 5′-UTR. The sequence similarity of the Pol sequences of *Tirant* and *Tirant_het* is approximately 80%, and the similarity of the repeat modules in 5′-UTR is 85% (Figure 3).

Thus, along with the specificity for a nucleotide integration target, the ZAM subgroup elements have specificity for integration into the euchromatin/heterochromatin that correlates with the structure of the regulatory region in 5′-UTR. Tandem repeats in the 5′-UTR of *Tirant* errantivirus seem to have been captured in the host genome. Possibly, targeted integration into the active chromatin allows the retrotransposon to escape from host defenses. Many viruses clearly have acquired accessory genes and regulatory sequences from their hosts. In particular, lentiviruses contain accessory genes that antagonize or circumvent host restriction factors [35].

## 4. Specific Terminal Nucleotides of Errantivirus Long Terminal Repeats Are Involved in the Interaction with Integrase

**Figure 4:**
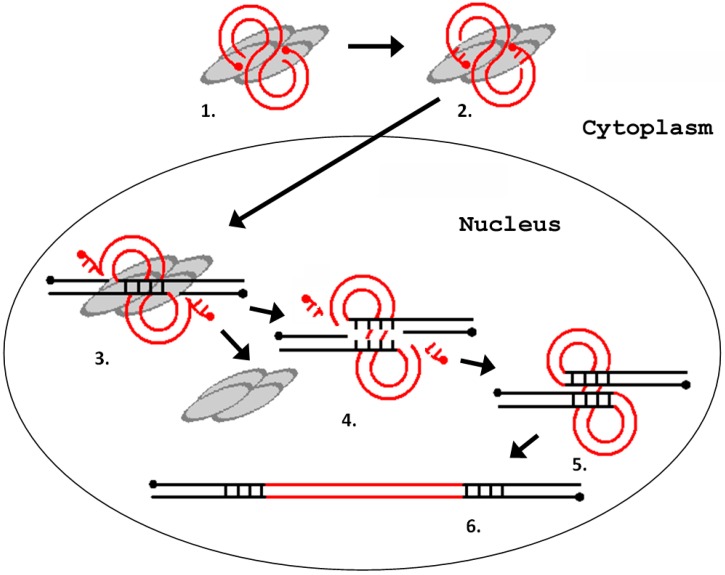
Schematic representation of the process of integration of a retrovirus (LTR retrotransposon) into the host genome. (**1**) interaction of integrase with a blunt-ended DNA substrate (other proteins are not shown); (**2**) removal of two terminal nucleotides from the 3′-ends of the DNA substrate (3′-end processing); (**3**) cleavage of the integration site; (**4**) removal of unpaired nucleotides at the 5′ ends of the DNA substrate; (**5**) filling-in of the gaps of the target DNA and ligation of discontinuities; and (**6**) repeating of the provirus integration site. The gray ovals represent integrase monomers. The red lines represent viral DNA, and the black lines represent chromosomal DNA. The dots indicate 5′-ends of the DNA.

The integration process can be divided into the following steps: (1) processing of the LTR ends; (2) recognition and cutting of target DNA in the host genome; and (3) integration of the LTR sequences into the target DNA [36]. All three steps are catalyzed by a retroviral integrase. Integrase is a part of the preintegration complex that recognizes the nucleotide sequences at the ends of the LTRs and prepares them for integration by removing the TG dinucleotide at the 3′-terminus of each chain (reaction of the 3′-end processing). The integration scheme is represented in Figure 4.

**Figure 5:**
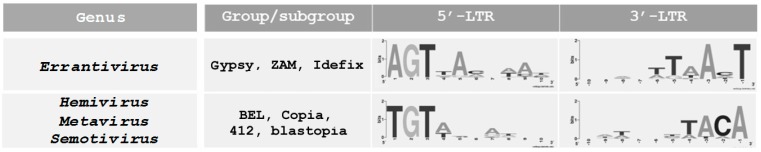
Multiple alignment of the 5′- and 3′-terminal sequences of the *D. melanogaster* LTR retrotransposons. The LTR retrotransposons of *D. melanogaster* can be divided into two groups depending on the composition of the end sequences [25]. Visualization performed using WEBLOGO [29].

All LTR-retroelements (LTR retrotransposons and retroviruses) obligatorily have inverted dinucleotides at the ends. Vertebrate proviruses have conserved 5′-TG/CA-3′ dinucleotides at the ends. It is believed that they are specific recognition sites for integrase and are a signal for 3′-end processing [37]. The protruding ends formed after processing comprise two terminal CA nucleotides that interact with the integrase. It has been shown that 12–15 subterminal nucleotides, in addition to the CA dinucleotide, can be employed in conjunction with the integrase [38]. The occurrence of 5′-TG/CA-3′ dinucleotides at the retrotransposon ends can be explained by the fact that TG, CA, and TA dinucleotides are the most deformable links in a DNA structure and are capable of local bending of the double helix due to the low energies of stacking interactions. Therefore, these three dinucleotides are often recognition sites for proteins that are involved in recombination, replication, and insertional events [39]. However, one exception is the integrase of *Drosophila* errantiviruses. According to an analysis of the terminal sequences, *D. melanogaster* LTR retrotransposons can be subdivided into two groups (Figure 5). The dinucleotides, TG/CA, are present at the ends of LTR retrotransposons of the BEL, Copia, and Gypsy groups of the *Metavirus* genus. However, errantiviruses (subgroups of *gypsy*, *ZAM*, and *Idefix*) and their derivatives have AGT/AnT trinucleotides at the ends, where “n” is usually A or C. In five of the eleven errantiviruses, all three terminal nucleotides are completely complementary; moreover, the errantiviruses, *Tirant*, *opus*, and *ZAM*, have five, six, and seven completely complementary nucleotides at the ends, respectively [25]. It is unclear how many and which nucleotides of the errantivirus ends are involved in the integrase and processing interactions.

## 5. LTR Retrotransposons of the *Metavirus* Genus Have a Chromodomain in the Integrase Structure

For obvious reasons, the most studied retroviral integrase is HIV-1 integrase. However, despite numerous attempts to establish an accurate pattern of DNA–protein interactions, the exact interaction mechanism of integrase with the target DNA is poorly understood [40]. Even the spatial structure of integrase is still uncertain. There are three domains in the integrase structure: N-terminal, central catalytic, and C-terminal [41]. Specific binding is obviously carried out by the most conservative central domain [42]. The role of the N-terminal domain during the process of integration is the least clear. This region contains a His-His-Cys-Cys motif, which is characteristic for the majority of retroviral integrases [43]. This domain appears to be involved in protein dimerization; its role in the binding with DNA is not significant. A mutant enzyme in which the N-terminal domain or HHCC-motif is absent loses the ability to carry out 3′-end processing and strand transfer [44]. The C-terminal is believed to participate in nonspecific binding of DNA [45].

In some LTR retrotransposons of the Gypsy group, including many chromoviruses of plants, algae, and fungi (but not yeast), the chromodomain is localized in the C-terminal domain of integrase and plays an important role in the interaction with the LTRs [10]. This domain is characterized by a conserved GPY/F motif. It is believed that this domain facilitates the interaction of integrase with chromatin. Chromodomains are found in integrases of vertebrate retroviruses of class 1, i.e., gamma- and epsilonretroviruses, including MLV. Of note, the GPY/F motif is present in *D. melanogaster* LTR retrotransposons of the *Metavirus* genus in the representatives of the two subgroups, 412 and blastopia [25]. Errantivirus integrases do not have a GPY/F motif. 

## 6. LTR Retrotransposons of the *Metavirus* Genus Are Able to Transfer Horizontally

The main difference between retroviruses and LTR retrotransposons is the presence of the *env* gene, which is responsible for infectivity. It is believed that *Drosophila* LTR retrotransposons of the Gypsy group initially had two ORFs. Then, they acquired the *env* gene from baculoviruses and, therefore, their infective properties [46]. However, in *Drosophila*, besides errantiviruses, an additional LTR retrotransposon has the *env* gene, *roo*. It is the LTR retrotransposon of the BEL group with one ORF (Figure 1). Meanwhile, the *env* genes of errantiviruses and the *roo* LTR retrotransposon are homologous; therefore, they have a common origin. It is shown that the acquisition of the *env* gene by the *roo* LTR retrotransposon occurred after the separation of Drosophilidae into a separate evolutionary branch of insects. Thus, errantiviruses may be the source of the *env* gene used by the *roo* element [46]. LTR retrotransposons of the *Drosophila* Copia group do not have the *env* gene. However, this does not mean that the appearance of *env* as part of the retroelement is impossible. Thus far, the only case of LTR retrotransposon of the Copia group with the *env* gene is the *SIRE* retroelement, which has been described only in soybeans [47].

Of note, LTR retrotransposons of the 412 subgroup have substantially identical copies in the genomes of different species of *Drosophila* and very close homologs (the identity in amino sequences of reverse transcriptase is more than 90%) in a very distant species (melanogaster, willistoni, virilis, and replete groups) [9]. This implies that LTR retrotransposons of the 412 subgroup can horizontally transfer without their own *env* gene. The question of how these elements move between species remains open: either they do not need the *env* gene function for infection, or the elements of this subgroup use a foreign envelope protein to move. The most likely possibility is a transmission of the retrotransposons through pseudotyping with envelope glycoproteins derived from errantiviruses. The presence of close homologs of 412 in the genomes of different species of *Drosophila* is correlated with the presence of close homologs of *gypsy* and *springer* errantiviruses in the genomes of the same species [9]. This does not preclude that 412 LTR retrotransposon uses the errantivirus *env* gene function for movement.

## 7. Consequences of the Retroelement Transposition

For a long time, it was believed that mobile elements are genomic parasites and nature removes them from participation in the functioning of the genome via the heterochromatization of sites where they are localized. However, recent molecular studies have shown that mobile element sequences, including retroelements, may acquire functional significance for the host genome during the course of evolution. The DNA sequence of any retroelement (retrotransposon or retrovirus) incorporated in the gene eventually accumulates mutations and degrades. Meanwhile, certain genes or regulatory sequences from the retroelement can be stored and undergo domestication and/or exaptation (change of function). As a rule, retroelement gene function is adapted to benefit the host genome. Thus, domestication of heterologous genes, including genes of retroelements, is one of the mechanisms of gene origin. The domestication of *gag* and *env* genes deserves special attention. Obviously, their functions can be adapted to protect the host genome from a retroviral infection via competition with homologous viral gene products. Some examples, known as the mammalian homologs of *gag* and *env* genes, participate not only in protection against viral infection but also in the control of cell division, apoptosis, placenta functioning, and other biological processes [48,49,50]. Therefore, “the scope” of domesticated capsid and envelope proteins could be much wider than previously thought and requires further study. *D. melanogaster* could be a good model for such research because its genome contains both *gag* and *env* homologs. It has been shown that both genes are under strong selection [51,52]. Currently, their functions are being actively studied, and it is possible that both genes are involved in the defense against viral infections.

## 8. Conclusions

The interaction of integrase with a target DNA sequence is a process dependent on the “complementarity” of DNA-binding domain of enzyme and DNA region that it connects. Mostly, three factors can influence on integration process: host chromatin status; genomic features such as histone modifications and transcription factor binding sites; and primary sequence of a target DNA. Specificity of vertebrate retrovirus integration into a target site is rather relative. The search for retroviruses and LTR-retrotransposons specifically integrating into a target is of great interest for the studies concerning the use of a site-directed mutagenesis.

Errantiviruses specifically integrate into the target DNA. In addition, tandem repeats in the 5′-UTR of *Tirant* errantivirus seem to direct its euchromatic localization. The integration specificity correlates with the structural features of the target DNA and the distinctive sequence of errantivirus LTR terminal nucleotides. The end sequences of LTRs in “nonspecific” LTR-retrotransposons of *Drosophila* (GT/CA dinucleotides) are, like in vertebrate retroviruses, highly conservative. It is believed that these dinucleotides have a low energy of stacking interaction and are, therefore, the most deformable links in the DNA structure, which are capable of forming a local bending to promote integration. In some “nonspecific” LTR retrotransposons of the Gypsy group (subgroups 412 and blastopia), chromodomain, which is localized in the C-terminal domain of integrase, probably plays an important role in the interaction with LTRs.

LTR retrotransposons of *D. melanogaster*, especially representatives of the Gypsy group, clearly demonstrate the possibility of mobile element evolution, which is based not only on their high rate of diversification but also on the ability to acquire individual modules or genes. The molecular rearrangement, transposition, recombination, and horizontal transfer, coupled with the selection of viable and adaptive variants of newly formed retrotransposons, play a key role in the evolution of retrotransposons and retroviruses. As a result of these changes, some retrotransposons or retroviruses acquire specific opportunities to integrate into actively transcribed regions of the genome, which is important for their future activity. The lack of molecular barriers for recombination between genes (or their fragments) can lead to multidirectional pathways of retroelement evolution followed by diversification of mechanisms of retroelement integration.
